# Applications, challenges, and strategies in the use of nanoparticles as feed additives in equine nutrition

**DOI:** 10.14202/vetworld.2020.1685-1696

**Published:** 2020-08-26

**Authors:** P. Ravi Kanth Reddy, Duvvuru Yasaswini, P. Pandu Ranga Reddy, Mohamed Zeineldin, M. J. Adegbeye, Iqbal Hyder

**Affiliations:** 1 Veterinary Dispensary, Taticherla, Andhra Pradesh, India; 2 Department of Veterinary Medicine, College of Veterinary Science, Sri Venkateswara Veterinary University, Tirupati, India; 3 Livestock Farm Complex, College of Veterinary Science, Sri Venkateswara Veterinary University, Proddatur, Andhra Pradesh, India; 4 Institute for Genomic Biology, University of Illinois at Urbana-Champaign, USA; 5 Department of Animal Medicine, College of Veterinary Medicine, Benha University, Benha, Egypt; 6 Department of Animal Science, College of Agriculture, Joseph Ayo Babalola University, Ikeji-Arakeji, Nigeria; 7 Department of Veterinary Physiology, NTR College of Veterinary Science, Sri Venkateswara Veterinary University, Gannavaram, India; 8 Department of Biotechnology, Institute of Farm Animal Genetics, Friedrich Loeffler Institute, Neustadt, Hannover, Germany

**Keywords:** antimicrobial, drug delivery, equine nutrition, feed additives, nano-minerals, nanoparticles

## Abstract

The rapid expansion of nanotechnology has been transforming the food industry by increasing market share and expenditure. Although nanotechnology offers promising benefits as feed additives, their usage in equines is primarily geared toward immunotherapy, hyper-immunization techniques, drug delivery systems, grooming activities, and therapeutic purposes. Nanoparticles could be engaged as alternatives for antibiotic feed additives to prevent foal diarrhea. Gold nanoparticles are proved to provide beneficial effects for racehorses by healing joint and tendon injuries. Because of the poor bioavailability of micro-sized mineral salts, the usage of nano-minerals is highly encourageable to improve the performance of racehorses. Nano-Vitamin E and enzyme CoQ10 for equines are no longer a simple research topic because of the increased commercial availability. Employing nanotechnology-based preservatives may offer a promising alternative to other conventional preservatives in preserving the quality of equine feed items, even during an extended storage period. While nanoparticles as feed additives may provide multitudinous benefits on equines, they could elicit allergic or toxic responses in case of improper synthesis aids or inappropriate dosages. The safety of nano-feed additives remains uninvestigated and necessitates the additional risk assessment, especially during their usage for a prolonged period. To adopt nano-feed additives in horses, there is an extreme paucity of information regarding the validity of various levels or forms of nanoparticles. Further, the currently available toxicological database on the topic of nano-feed additives is not at all related to equines and even inadequate for other livestock species. This review aims to provide new insights into possible future research pertaining to the usage of nano-feed additives in equines.

## Introduction

Nanoparticles are either natural or synthetic substances covering a minimum of 50% proportion with a size ranging from 1 to 100 nanometers [1]. However, the standard 50% proportion is flexible and can be lowered under a few inevitable circumstances, thus, causing a negative impact on the animals’ health. The naturally available nanoparticles are so ubiquitous that their presence can be discerned in a wide range of biological matter [2].

The ever exploration of science and technology has extended the usage of nanoparticles in different fields of science. Although the usage of antibiotics as a feed additive in various livestock species is advantageous in decreasing the bacterial load, the development of microbial resistance to various antibiotics led to a ban on their usage by European Union in 2006. Because of the bactericidal properties, nanoparticles such as nano-zinc and nano-silver could serve as potential alternatives for antibiotic feed additives. Besides, the usage of nanoparticles as feed additives instead of their macro-counterparts reduces the mineral excretion and environmental pollution [3]. The total dosage required to reach effective concentrations in serum is lower at nano levels compared to their non-nano counterparts. For instance, feeding nano-zinc oxide (ZnO) at 800 mg/kg improved intestinal morphology, average daily gain, and plasma zinc level, similar to the non-nano-ZnO at 3000 mg/kg [4].

The elaborative review by Elghandour *et al*. [5] advised the cautious incorporation of feed additives in equine nutrition. Few studies have been conducted on livestock species, including ruminants [6], poultry [7], swine [8], and rabbit [9]; however, the research conducted on the horses is scanty as per the literature collected. The results from any of the aforementioned species might not be approved in equines due to the variations in vulnerability levels, pharmacodynamics, and pharmacokinetics compared with other livestock species. This review aims to collate the available information about the nanoparticle-based supplements and determine the possibility, benefits, threats, and challenges of using nanoparticles as feed additives in equine nutrition.

### Nanoparticles as feed additives

**Figure-1:**
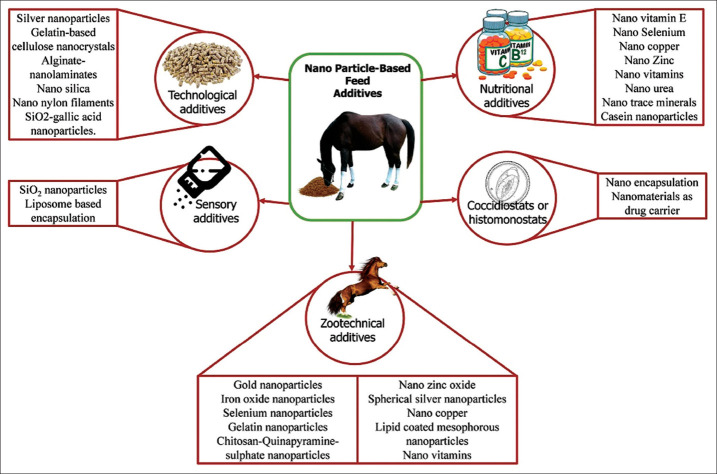
List of nanotechnology-based feed additives in equine nutrition. [Source: Duvvuru Yasaswini prepared the figure using Adobe Photoshop].

An investigation of the current literature on nanoparticles showed that the nanomaterials were mainly used in equines as drug delivery systems and therapeutics [3,5,10,11]. Indeed, as per the literature collected, no research was available on the direct usage of nanomaterials as feed additives in equine. Based on their function and sort of action, the nanoparticle feed additives were classified, as shown in Figure-1. The source, methodology, and findings of nanotechnology-based research works in equines are presented in Table-1.

**Table-1:** Source, methodology, and findings of nanotechnology-based on research work in equines.

Nanoparticle	Source of research	Species	Period administered	Methodology	Findings of the research work
Gold nanoparticles	[17]	*In vitro*	*In vitro* incubation for 24 h	Equine whole blood was incubated with 0.1 mL, 0.5 mL, and 1mL of gold nanoparticles-stabilized PVP solutions	Secretion of one morphogenetic protein 2, vascular endothelial growth factor, and fibroblast growth factor 1. Decreased concentration of Interleukin-1 alpha
GNP-CpGO	[21]	Horse	Inhaled thrice with 2-day intervals	GNP-CpGO nebulized to both healthy and RAO horses	Increased expression of anti-inflammatory and anti-allergic cytokines Decreased allergic condition
GNP-CpGO	[22]	Horse	Inhaled 5 times with 2-day intervals	Twenty-four horses suffering from chronic RAO were inhaled with placebo or GNP-CpGO Clinical examination was done before administration, immediately after final inhalation and 4-weeks after final inhalation	Decreased nasal discharge, respiratory effort, tracheal secretion, and neutrophil count. Increased arterial oxygen pressure
GNP-CpGO	[23]	Horse	Inhaled 7 times with 1-day interval	Twenty horses suffering from asthma were treated either with GNP-CpGO alone or CpG-GNP with allergens. Clinical examination was done before administration, immediately after final inhalation and 6-weeks after final inhalation	Improved partial oxygen pressure, breathing rate, nasal discharge, viscosity and amount of secretion, and neutrophil percentage
GNP-CpGO	[20]	Horse	The lavage fluid was incubated with 0.275 mg GNPs loaded with or without 13.5 mg GNP-CpGO	Bronchoalveolar lavage fluid collected from horse suffering from RAO	Stimulated release of interleukin-10 from equine alveolar lymphocytes
Isometamidium hydrochloride- sodium alginate/gum acacia nanoparticles	[59]	Horse	Peripheral blood mono nuclear cell cultures were treated with ISMM-SA NPs and incubated for 24 h	Peripheral blood collected from Marwari horse	The cytotoxic effects of drug were predominant on treating with plain Isometamidium compared to those with sodium alginate based nanoparticles
L-M-SNP	[28]	Mice	1 mg ML336 loaded L-M-SNP was administered through intra peritoneal route for a period of 15 days	Spleen, lung, liver, kidneys, and brain were collected after euthanizing the mice	Reduced brain viral titer compared to phosphate buffer saline control
Nano silver	[49]	Horse	Disk diffusion assay for 20 h	Air sampling done from horse barn containing 108 horses	Antibacterial activity of nanosilver on airborne *Staphylococcus* spp from air collected from horse barn
Nano silver	[50]	Horse	Horse dung sampled 4 months a year Disk diffusion assay for 24 h.	Dung sampling done from horse barn containing 100 horses	Silver nanoparticles displayed strong antimicrobial activity against *Escherichia coli* with a minimum inhibitory concentration of 15 µg/mL
Nano silver	[18]	Donkey	28 days	Five wounded donkeys Clinical, microbiological, and histopathological estimation	Prompt granulation tissue development, reducing scar width, and higher epithelialization rate
Selenium nanoparticles	[15]	Donkey	10 days	Eight donkeys fed with a diet supplemented with either normal saline or SENPs at 0.5 mg/kg BW	Increased blood selenium concentration and HSP90 expression during post-exercise period
Selenium nanoparticles	[16]	Donkey	10 days	Eight donkeys fed with diet supplemented with either normal saline or SENPs at 0.5 mg/kg BW	Decreased creatinine and blood urea nitrogen at 72-h post-exercise recovery time
ZnO NPs	[55]	Horse	RBC suspension incubated at 37°C for 90 min dissolved in 10 mg ZnO NPs	RBC sample collected from Marwari horse	Revealed a concentration dependent clustering of horse erythrocytes
ZnO NPs loaded with or without sodium alginate-gum acacia hydrogels	[55]	Horse	RBC suspension incubated at 37°C for 90 min dissolved in polymerized ZnO Nps	RBC sample collected from Marwari horse	Administering polymerized nanoparticles reduced RBC agglomeration and oxidative stress of horse RBC

GNP=Gelatin nanoparticle, L-M-SNP=Lipid-coated mesoporous silica nanoparticle, RBC=Red blood cell, ZnO NPs: Zinc oxide nanoparticles, PVP=Polyvinylpyrrolidone

### Nanoparticles as zoo-technical additives

Zoo-technical feed additives favorably affect the performance of horse. This group includes gut flora stabilizers, digestibility enhancers, and substances that affect the environment, and other additives, which improve the nutrient status in equine. The beneficial effects of antibiotics as zoo-technical feed additives in various livestock species could not be assured in equine because of the dissimilarities in dosage requirements [5]. Nanotechnology may be viewed as a potential alternative against various antibiotic feed additives due to the ability of metal nanoparticles’ in lysing the Gram-positive and Gram-negative bacterial cell walls [11]. The foal diarrhea is one of the most common causes of reduced growth performance and increased susceptibility to severe infectious diseases. The bacteria such as *Clostridium perfringens*, *Clostridium difficile*, *Escherichia coli*, *Bacteroides fragilis*, *Enterococcus*, and *Aeromonas* spp. are the common etiological agents for foal diarrhea [12]. Dietary supplementation of nano-ZnO at 800 mg/kg decreased diarrhea rate and improved average daily weight gain [13]. Similar to nano-ZnO, spherical silver nanoparticles are also known to decrease the incidence of diarrhea, due to their bacteriostatic and bactericidal effects on *Salmonella* and *Shigella* spp. [13]. Although neither of the two studies was conducted in equines, these results could be adopted to foals, thereby promoting the usage of nano-ZnO and nano-silver particles as feed additives. Supplementation of nano-copper is well proven to minimize or prevent post-weaning diarrhea in swine; however, no studies were reported in equine. Further, the abnormal relapse of infection led diarrhea, even after recovery from the initial incidence of disease, necessitates the usage of nano-compounds as sustained antimicrobial feed additives in foals [12].

Application of static magnetic field to equine adipose-derived mesenchymal stem cells pre-incubated with iron oxide nanoparticles could drive the nanoparticles to any desired site of the body [14]. These findings project the application of iron oxide nanoparticles for tissue regeneration in case of equine injuries such as bone fractures. Because of the multipotent and self-renewal capacity of mesenchymal stem cells, iron oxide nanoparticles are variably used in regenerative medicine and tissue engineering. Oral administration of selenium nanoparticles (SeNPs) at 0.5 mg/kg BW of donkey, for 10 consecutive days, resulted in a significantly increased blood selenium concentration and HSP90 expression during the post-exercise period [15]. In a similarly designed study, the SeNPs administration was able to decrease creatinine and blood urea nitrogen at 72-h post-exercise recovery time. The authors attributed these positive effects to the ability of Se incorporation into selenocysteine and prevention of tissue oxidation damages [16]. These two studies may elucidate the favorable role of SeNPs in combating the stressful conditions in equine species. Further, gold nanoparticles were proved to activate platelets and increase the secretion of morphogenetic protein 2, vascular endothelial growth factor, and fibroblast growth factor 1 in equines [17]. These results highlight the importance of gold nanoparticles in equine regenerative medicine, particularly for racehorses, which are more susceptible to ligament, tendon, and joint injuries.

More recently, a comparative study on antimicrobial effects of silver nanoparticles with amoxicillin/metronidazole mixture showed the superiority of nanoparticles in wound healing through prompt granulation tissue development, reducing scar width, and along with higher epithelialization rate [18]. The silver nanoparticles are also used as additives in the fabric of equine’s bandage as a part of wound dressing to improve the mechanical potential and accelerate the healing rate [19]. These bandages are being sold under different trade names in equine markets, claiming a reduced wound inflammation, biofilm-complications, and count of odor causing bacteria along with improved healing activity.

Because of the superior carrying capacity, protection ability, sustained-release property, and target tissue reaching ability, nanoparticles are extensively used as drug delivery systems in horse. Fuchs *et al*. [20] proposed the gelatin nanoparticles (GNPs) based on drug delivery system as a novel system for immunotherapy in horse-related preformulation studies. Employing GNP-based Cytosine-Phosphate-Guanine-Oligodeoxynucleotides (GNP-CpGO) delivery system maximized the efficacy of immunotherapy in horses suffering from recurrent airway obstruction (RAO) [21]. Later, the same authors tested the effectiveness of GNP-CpGO in relieving the symptoms of chronic RAO in a placebo-controlled and randomized clinical trial. The nanoparticles were able to decrease nasal discharge, respiratory effort, tracheal secretion, and neutrophil count along with an increase in arterial oxygen pressure [22]. In a continuation work, they also proved that CpG-GNP application is efficient in improving the partial oxygen pressure, breathing rate, nasal discharge, viscosity and amount of secretion, and neutrophil percentage [23]. The authors also claimed the future applicability of nanocarrier-mediated immunotherapy to humans suffering from asthma. Because of the increased popularity of GNP-CpGO in treating RAO in equines, Geh paved a way to produce large quantities of GNPs in a reproducible quality by a one-step desolvation technique [24].

Although chitosan nanoparticles are promoted as growth promoters and feed additives in poultry species, their usage in horses is restricted to the hyper-immunization technique for anti-venom preparation [25]. Horses are extremely sensitive to quinapyramine sulfate (QS), a trypanocidal drug used against surra. In this regard, formulating chitosan nanoparticle-based QS may sustain the drug’s release, thus reducing the side effects in horses [26,27]. A recent study conducted in mice revealed the lipid-coated mesoporous silica nanoparticle as an effective carrier for ML-336, which is a chemical inhibitor of Venezuelan equine encephalitis virus [28].

Nowadays, nanotechnology is also engaged in boosting grooming results. These grooming solutions contain nano vitamins and minerals as key ingredients, which are easily absorbed into the horse’s skin, thus providing a natural shine. Sub-optimal levels of copper and zinc may cause changes in color of the horse’s hair coat. Feeding copper and zinc nanoparticles can help in darkening the horse mane by preventing sun bleaching. Few companies are specifically formulating the plant-extract based nano-suspensions to use as topical agents. For instance, Audevard Laboratories designed a plant-extract based nano-product (Flymax) in spray form to prevent tick-borne diseases such as equine piroplasmosis. Chromium nanoparticles are known to increase leanness in poultry and swine meat by lowering cholesterol and increasing muscle protein content; nevertheless, these results are yet to be tested in horses [29,30].

### Nanoparticles as nutritional additives

Nutritional additives provide specific nutrients to animals. The group’s major elements include amino acids, vitamins, trace minerals, and polyunsaturated fatty acids, which are intended for direct consumption in small measured amounts. The nanoparticles are being explored as nutraceutical delivery systems in the food industry [31]. Authors recommend reading two elegant reviews by Singh *et al*. and Sekhon, which dealt with the nanotechnology applications in food and food processing industries [32,33]. Designing a carrier nanoparticle for administering additional nutrients in equines is a challenging task because of the unique environment and pH levels of the gastrointestinal tract. The nano-carriers must surpass all these difficulties to deliver the nutrients at a small intestine level for efficient absorption. Due to their emulsifying and good gelling properties, proteins are considered as generally recognized as safe (GRAS) category of nano-carriers. A gastrointestinal tract-simulated *in vitro* release study showed canola protein cruciferin as successful carrier material for beta-carotene, which was released at intestinal pH [34].

Feeding processed (rolled, extruded, and micronized) cereal grains may cause loss of several minerals or vitamins, thus predisposing horses to various metabolic disorders. The bioavailability is directly proportional to bioaccessibility, absorption, and molecular transformation [35], which is either improved or positively altered by nanotechnology. Thus, nanomaterial-based delivery systems increase the bioavailability of nutritional compounds. The physiological requirement of nutritional additives is high in heavy working and racehorses. The inorganic mineral salts show poor bioavailability due to other coelements, which inhibit the absorption. Thus, the incorporation of inorganic minerals in equine feeds increases their excretory rates and poses an environmental risk. The application of nano-sized minerals may increase bioavailability with a consecutive reduction of the excreted contents. The hypothesis behind the improved growth or production performance with mineral nanoparticles is related to an enhanced absorption rate of nutrients. The increased surface area and reduced size of mineral-nanoparticles improve certain physicochemical properties, therefore, extending their applications to many fields [36]. The technique of nano-encapsulation is highly beneficial in delivering Vitamins A, C, and E to the body tissues. Apart from the increased absorption and diffusion efficiency, the nano-encapsulation technique aids in decreased inflammation and improved healing process. However, as per the literature collected, no research was ever conducted on exploring the efficacy of mineral-nanoparticles as feed additives in equines.

Nano form of Vitamin E is the most and probably one among very few nano-elements ever researched in equine nutrition. Consumption of enough Vitamin E is essential to support optimal neurological and muscular activity in performance horses. Several commercial products are available on nano-dispersion forms of Vitamin E in the equine market. These are generally recommended for pregnant or lactating mares, breeding stallions, racehorses, and those recovering from injury or illness. Coenzyme Q-10, a key nutrient for energy generation in mitochondria, is another nano-suspension available in equine markets for targeted nutrition. Apart from ubiquinone-deficient cases, the coenzyme Q-10 is highly beneficial in combatting the oxidative stress in racehorses. However, the bioavailability of CoQ10 is very low due to poor water solubility. The surfactant-based nano-suspensions are known to improve the solubility and bioavailability of CoQ10 enzyme components. Racehorses devoid of dietary CoQ10 supplementation could suffer from impaired health, ultimately exhibiting poor physical performance [37]. These components are also known to work synergistically with Vitamins C and E. Casein nanoparticles are known as effective carriers of hydrophobic nutrients and could facilitate nutrient delivery in weanlings, consequently increasing their growth rates [31]. Casein micelles are generally considered as natural nanoparticles and bind with proteins, calcium, and other nutrients to allow for transport from mare to foal.

Surprisingly, horses can tolerate urea containing ruminant feeds such as molasses blocks and dry licks up to a certain extent. The non-protein nitrogen compounds could be used as feed additives for equines because of the urease activity in horse cecum, which is about 25% that of the rumen [38]. However, it is noteworthy that horses are sensitive to urea with a narrow threshold margin, and even a tiny dose above the threshold may result in devastating effects. Although not recommended, feeding urea to mature horses on all forage diet and marginal protein intake may result in beneficial effects regarding feed cost [39]. In this regard, reducing the urea solubility may cause its sustained release [40,41], accordingly decreasing the release and excretion rates. A scientific study proved a reduced release of urea by incorporating it in the hydroxyapatite nanoparticles [42], which could be used in mature horses devoid of protein-based food.

### Nanoparticles as technological additives

Technological additives improve the hygiene and quality characteristics of equine feed or supplements. The group includes antioxidants, preservatives, emulsifiers, stabilizing agents, silage additives, and acidity regulators. Of late, the investigation of nanoparticles as biocides to preserve foodstuffs is of growing interest [31]. The contamination of equine feed storage bins with pathogens, especially salmonella, may cause devastating effects on equine well-being [43]. Using silver nanoparticles-implanted plastic bins could minimize the animals’ health risks due to their antimicrobial property [44]. Metal-based nanoparticles are assumed to migrate from storage tins; however, recent research works revealed no detectable levels of silver nanoparticles in impregnated feed containers. Hence, more than a dozen silver-based zeolites were approved by United States Food and Drug Administration (USFDA) for disinfection purpose [45]. A German study revealed that cocontamination with mycotoxins is very common in horse feed bought from commercial markets. Although the mycotoxin concentrations in the study were below the threshold level of toxicity, they cause skin allergies, inflammation, and may even pose serious consequences in severe cases. In this aspect, addition of carbon-based and polymeric nanoparticles to mycotoxin contaminated feed may decrease or completely prevent the toxicity due to their higher binding capacity with toxins [46].

Propionic acid is a common preservative added to horse feeds to reduce dust and mold, consequently increasing the shelf life. Although not problematic, the horse owners are being known to raise several concerns over the purchase of feed preserved with propionic acid. Employing nanotechnology-based preservatives, namely, gelatin-based cellulose nanocrystals, chitosan films with nano-SiO_2_, alginate nano-laminate coating, and nano-silica overlays may offer a promising alternative to other conventional preservatives in preserving the quality of fresh feed items, even during extended storage period [47]. Furthermore, aluminum, the most commonly used anticaking agent in horse feeds, is harmful to horses because of its inhibitory effects on phosphorus absorption. Developing any alternatives for the standard anticaking agents in horse feeds are need of the hour. Recently, European Union has registered the SiO_2_ particles in nano-size range as anticaking agents [48]; however, these particles have to be validated in equine feeds. Although most of the nano-metal particles are known to produce oxidative stress by producing reactive oxygen species, few reactive nanomaterials such as polymer coatings and SiO_2_-gallic acid are being used in the food industry as antioxidants carriers [46]. The performance of antioxidants could be improved by adopting lipid-based nanoencapsulation systems, which aid in increased solubility and bioavailability of the particles [49]. Nanoparticles could stabilize various bioactive components by preventing their degradation in the feed. This technique could be used to preserve the volatile bioactive components, which are known to provide beneficial effects on the health and well-being of horses [5].

Pathogen entry by direct infection can be curtailed by strict biosecurity measures; however, air, water, and equipment may provide accession points for pathogen entry. The micro-environment of horse barn is generally loaded with various pathogenic microbes, thereby causing negative effects on equine welfare and even health of the workers in the horse stable. The silver nanostructures were found as effective antimicrobial compounds against *Staphylococcus* spp. and *E. coli* isolated from barn air and horse dung, respectively. In this context, the authors testified the usage of silver nanostructures in suspension form as a component for sanitizing, cleaning, and disinfecting the horse stables [49,50]. However, promoting these antimicrobial nanoparticles as direct feed additives in equines require *in vivo* studies involving enumeration of fecal pathogenic counts and recordings of growth and other production parameters. Further, the nano-nylon filaments embedded with minerals are generally sold as rugs for exercise horses. These rugs are known to provide infrared therapy; hence, reduce fatigue, swelling, and muscle tension in horses, consequently speeding up the recovery time.

### Nanoparticles as sensory additives

Sensory additives are meant to improve the appetite and palatability of feed. Flavor enhances the palatability and appetite of the animal by delivering sensory perception of taste and smell. The commercial horse feed industry has been using food flavors to overcome feed neophobia and encourage the intake of unpalatable supplements, water, and anthelmintic drugs. Goodwin *et al*. [51] reported fenugreek and banana flavors as the most desirable flavors to promote the intake of unpalatable pellets. Although never tested in equine, several nano-encapsulation techniques have been successfully used to improve the flavor release in food products (Regulations, EC, 133). The ability of SiO_2_ nanomaterials to carry fragrances or flavors in food products is well proved [52]. According to a study conducted by Malheiros *et al.*, nano-based liposome entrapment technique has a great advantage in delivering the flavors effectively, when compared to other encapsulation technologies [53]. Usage of the liposome-encapsulation technique is necessary to deliver essential flavors in horse feeds.

### Nanoparticles as coccidiostats and histomonostats

**Figure-2:**
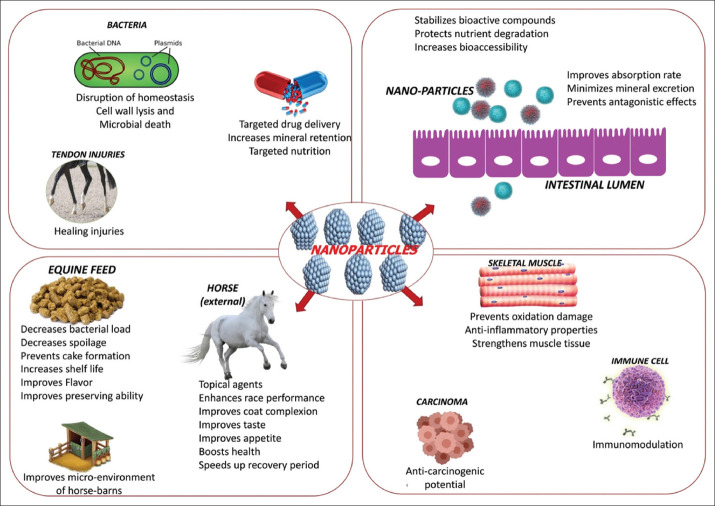
Different modes of action of various nanotachnology-based feed additives in equine nutrition. [Source: Duvvuru Yasaswini prepared the figure using Adobe Photoshop].

Coccidiostats or histomonostats are the agents used to control animal health. The use of coccidiostats and histomonostats as feed additives is primarily limited to livestock species other than equine. Recently, Dubey and Bauer reviewed the worldwide information on prevalence, pathogenesis, and epidemiology of Eimeria infections in equids [54]. They reported that Eimeria infections are known to cause acute illness and sudden deaths in foals. The common coccidiostats in poultry or calves’ diets, such as monensin and lasalocid, are extremely toxic to equine. As per the current literature, no nanoparticles were ever used to treat equine coccidiosis. However, the growing awareness on the benefits of nanotechnology could promote its usage as either drug carrier or therapeutic agent in the treatment of coccidiosis and other protozoal infections in equines. The pictorial representation of different modes of action of various nanotechnology-based feed additives in equine nutrition is presented in Figure-2.

## Challenges and strategies

**Figure-3:**
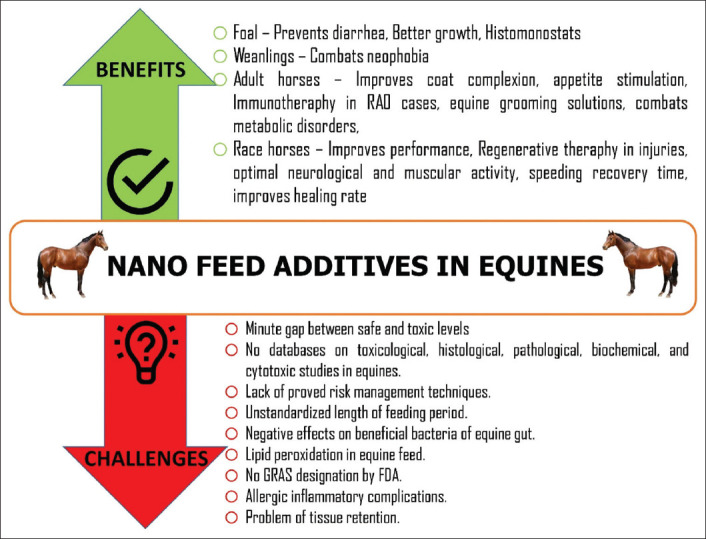
Benefits and challenges of administration of nanotachnology-based feed additives in equine nutrition. [Source: Duvvuru Yasaswini prepared the figure using Adobe Photoshop].

This review so far discussed the health benefits of nanoparticles in horses, with an emphasis on their antimicrobial activities and performance-enhancing effects. Addressing the challenges, constraints, and their remedies in the usage of nano-feed additives in equine are essential to complete the review. The benefits and challenges of the administration of nanotechnology-based feed additives in equine nutrition are presented in Figure-3. It was only a few years ago that nano-feed additives were considered as highly beneficial for animal health, and the principal research areas were the method of preparation, encapsulation techniques, chemical structures, and therapeutic applications. Nanomaterials are now being recognized as feed additives with potential health benefits, but the research is limited to livestock species other than equines.

Employing default assumptions and conclusions for the nano-feed additive usage may cause adverse effects, because of the minute gap between safe and toxic levels. Being under the defined and unexplored subject, arriving a safe dosage of nano-feed additive in equines, at the field level, requires an extensive investigation. The interaction of nanoparticles with various biological components should be well-reviewed while administering as a feed additive. In this regard, evaluation of the hemolytic potential of ZnO nanoparticles revealed a concentration-dependent clustering of horse erythrocytes [55]. The authors also claimed for complete investigation on interactions between horse red blood cells (RBC) and ZnO nanoparticles before promoting the particles’ usage in horses. Although the preliminary findings reveal no specified side effects, the nanoparticle application should always be associated with histological confirmation. Moreover, the uncertainties in the results of various studies are extreme in animal nutrition-related research works. Hence, the final dosages should be arrived after conducting meta-analysis or any other equivalent statistical analysis, which combines the results of diverse scientific studies. In a review, Swain *et al*. opined that the actual bioavailability of few mineral-based nanoparticles, such as ZnO nanoparticles, is still to be specified accurately [56].

The affirmative results of *in vitro* works could be applied to equines after their validation with several *in vivo* works. Unfortunately, very limited *in vivo* works exist on testing nanoparticles as feed additives in equine species. The apparatus used for validating the results is another crucial aspect to be considered. For instance, an *in vitro* study conducted to know the interactions between horse RBC and nano-ZnO revealed the absence of hemolysis by spectrophotometric method, while the phase-contrast microscopic examination reported a dose-dependent aggregation of erythrocytes [55].

At present, the equine stable owners and hostlers have a limited ability to characterize and manage risks associated with commercial use of nanomaterials. The extreme unspecified and indefinite problems posed by nanomaterials usage need to be familiarized by building new risk assessment-based tools. In an innovative study, Beaudrie and Kandlikar adopted a “horses for courses” approach using prevalent analytical tools such as multi-criteria decision analysis, risk ranking, and control banding approaches to arrive at a final decision regarding the nanoparticle usage [57]. According to their results, the innovations pertaining to nanotechnology are often made by small enterprises, which have neither the resources nor the expertise to assess the health, well-being, and environmental hazards completely. Further, the risk assessment procedure requires a vast number of factors, which include, but not limited to, pH of fluids, osmotic concentration, commensal microbes, physical forces, and the rate of absorption, distribution, metabolism, and excretion of nanoparticles.

Reduced size and increased surface area in nanoforms make them more toxic than their macro counterparts [58]. Apart from toxicity complication, excess of one mineral may also result in the deficiency of another due to antagonistic action [5]. Using polymers such as sodium alginate or gum acacia may prevent undesirable effects by providing an effective delivery system. In an analogous study, a comparison of naive ZnO NPs to those loaded with sodium alginate-gum acacia hydrogels revealed reduced oxidative stress and horse RBC agglomeration in polymeric ZnO NPs [55]; this phenomenon explains the beneficial effects of polymeric compounds. Further, the cytotoxic studies of isometamidium-sodium alginate nanoparticles on equine mononuclear and erythrocytes revealed the efficiency of sodium alginate in enhancing the efficacy at lower doses, while curtailing the undesirable side effects [59]. Nevertheless, the number of experiments on the cytotoxicity of sodium alginate nanoparticles in horses is largely inadequate, and further research is required before promoting their usage at extensive scales. It should also be remembered that the nanoform-toxicity relationship, as mentioned earlier, would not always show a positive correlation and might change with the type of particles involved. For example, the nano-silver colloidal solution is more stable at the stomach’s acidic pH with a lower ability of absorption by eukaryotic cells when compared to metallic silver forms. Hence, the nano-silver types are minimally toxic with greater antimicrobial efficiency [60]. Further, a practice of unempirical application of various nanomaterials, such as silver-nanoparticles, has been observed, which requires elaborative scientific information for monitoring their potential tolerance and environmental risk assessment [49,50].

Several metal-based nanoparticles are known to accumulate in body tissues on regular administration, even at minimal levels. Hence, the length of the feeding period is also an essential factor to be considered before promoting any nanotechnology-based feed additive. Chronic ingestion of silver compounds may accumulate in eyes, skin, and other vital organs such as liver. The accumulation-induced cytotoxicity effects of silver nanoparticles were well-reviewed by Akter *et al*. [61]. In this aspect, they are promoting the usage of nanoparticles with accumulative tendency as short-term feed additives are safe rather than their usage for prolonged periods.

The equine digestive system is very delicate so that the response is rapid in case of altered beneficial flora, especially *Lactobacillus equi* species. Apart from the anti-pathogenic and immunomodulatory effects, these bacteria are known to counter the pro-carcinogenic bodies produced in the course of certain enzymatic activities and digestive processes of the microbial flora within the horse colon [62]. The effects of antimicrobial nanoparticles on these beneficial bacteria are uncertain and have to be tested before employing them as equine feed additives. Another notable constraint is the inconsistency of results. An *in vitro* study conducted by Fondevila *et al*. revealed the harmful effects of silver nanoparticles against coliforms without affecting *Lactobacilli* species [60]. Whereas, Tian *et al*. [63] reported negative effects of the same components on *Lactobacilli* species without affecting other opportunistic pathogens such as *E. coli* and *Staphylococcus aureus*. The authors further reported that the susceptibility of *Lactobacillus* species to silver nanoparticles is due to the acidic environment created by lactic acid produced as a primary byproduct.

Amidst the growing demand for quality food and environmental stewardship techniques, researchers have been developing nano-packaging technologies in the food industry. Although their usage is limited to food manufacturing alone, in the near future, the practice may also become functional in equine feed industry. However, Echegoyen *et al*. raised a negative concern over the usage of nanocomposites for food-packaging applications [64]. They reported that the nanoparticles would migrate into the food from outer packaging, consequently resulting in unintentional consumer exposure. One more potential constraint regarding the nano-packaging is the release of metal ions into the food products, which further results in lipid peroxidation and DNA damage [47]. Among those nanomaterials, silver, copper oxide, and ZnO are the three commonly found metal-leaching nanoparticles [47].

The potential positive benefits acquired on supplementing nanoparticles to any species other than equine could not be adopted in the latter species [5]. Literature indicates that live yeast cell derivative stimulates epithelialization, angiogenesis, and collagen formation in dogs. While the same derivative caused delayed wound contraction, consequently prolonging the wound healing in horses [65]. Moreover, the currently available toxicological database regarding nano-feed additives is not at all related to equine and even inadequate for other livestock species. In this view, specific organizations such as US Environmental Protection Agency, FDA, International Organization for Standardization, and the Organization for Economic Cooperation and Development, as well as many regulatory bodies, have issued certain guidelines on potential risks posed by nano-feed additives in food animals. However, none of these guidance documents is directly applicable to equines.

Despite the novel properties of nano-metal particles, their safety on dietary supplementation is ever questionable. The GRAS designation for different metal particles specified by FDA is mostly suitable to micron size range; however, in nanotechnology, the metal forms are reduced to the nanoscale, which may pose toxicity [55]. Administration of a few nanoparticles may promote allergic pulmonary inflammation [47]. Intranasal exposure of mice to ovalbumin plus nano-silica particle resulted in allergen-specific Th_2_-type allergic immune responses [66].

The assessment of impending threats and risks of nano-feed additives should be facilitated by setting flawless and unambiguous criteria for their identification [67]. The tissue retention of nanoparticles is an initial phase for toxicity. Hence, any study conducted on the promotion of nanoparticles as feed additives should check the range of tissue retention. As long as not all the constraints mentioned above are addressed, the usage of the nano-feed additive remains inappropriate in equines.

## Conclusion

Manipulation of materials at nanoscale provides a wide range of properties, which could be beneficially used in equine nutrition. The literature on the usage of nanoparticles in horses is scanty. Supplementation of nanoparticles in equine diets may not always result in unambiguous outcomes. No limiting concentrations of nano-feed additives usage have been fixed in equines. Promoting any nanoparticle as feed additive requires a piece of concise information about the potential toxicity of the particular compounds against host. Further, the correlation between beneficial effects of different nano-feed additives and their concentrations has not yet been clarified, even in other livestock species. Appropriate characterization and toxicological data of nano-feed additives are prerequisites to permit their usage in equines. Follow-up studies should aim to extrapolate the nano-feed additives-based research to field level feedlot situations. Additional research should also focus on the long-term effects of nanoparticles before recommending them for practical application.

## Authors’ Contributions

PRKR: Conceptualized and designed the review. PRKR, DY, and PPRR: Drafted the manuscript. DY: Drawn the images. MZ, MJA, and IH: Collected the required literature. PPRR and IH: Finalized the manuscript. All the authors read and approved the final manuscript.

## Competing Interests

The authors declare that they have no competing interests.

## Publisher’s Note

Veterinary World remains neutral with regard to jurisdictional claims in published institutional affiliation.
